# Improving Patient Experience and Primary Care Quality for Patients With Complex Chronic Disease Using the Electronic Patient-Reported Outcomes Tool: Adopting Qualitative Methods Into a User-Centered Design Approach

**DOI:** 10.2196/resprot.5204

**Published:** 2016-02-18

**Authors:** Carolyn Steele Gray, Anum Irfan Khan, Kerry Kuluski, Ian McKillop, Sarah Sharpe, Arlene S Bierman, Renee F Lyons, Cheryl Cott

**Affiliations:** ^1^ Institute for Health Policy, Management and Evaluation Dalla Lana School of Public Health University of Toronto Toronto, ON Canada; ^2^ Bridgepoint Campus Lunenfeld-Tanenbaum Research Institute Sinai Health System Toronto, ON Canada; ^3^ School of Public Health and Health Systems University of Waterloo Waterloo, ON Canada; ^4^ Li Ka Shing Knowledge Institute St. Michael’s Hospital Toronto, ON Canada; ^5^ Department of Physical Therapy University of Toronto Toronto, ON Canada

**Keywords:** eHealth development, mobile apps, multi-morbidity, complex patients, primary care

## Abstract

**Background:**

Many mHealth technologies do not meet the needs of patients with complex chronic disease and disabilities (CCDDs) who are among the highest users of health systems worldwide. Furthermore, many of the development methodologies used in the creation of mHealth and eHealth technologies lack the ability to embrace users with CCDD in the specification process. This paper describes how we adopted and modified development techniques to create the electronic Patient-Reported Outcomes (ePRO) tool, a patient-centered mHealth solution to help improve primary health care for patients experiencing CCDD.

**Objective:**

This paper describes the design and development approach, specifically the process of incorporating qualitative research methods into user-centered design approaches to create the ePRO tool. Key lessons learned are offered as a guide for other eHealth and mHealth research and technology developers working with complex patient populations and their primary health care providers.

**Methods:**

Guided by user-centered design principles, interpretive descriptive qualitative research methods were adopted to capture user experiences through interviews and working groups. Consistent with interpretive descriptive methods, an iterative analysis technique was used to generate findings, which were then organized in relation to the tool design and function to help systematically inform modifications to the tool. User feedback captured and analyzed through this method was used to challenge the design and inform the iterative development of the tool.

**Results:**

Interviews with primary health care providers (n=7) and content experts (n=6), and four focus groups with patients and carers (n=14) along with a PICK analysis—Possible, Implementable, (to be) Challenged, (to be) Killed—guided development of the first prototype. The initial prototype was presented in three design working groups with patients/carers (n=5), providers (n=6), and experts (n=5). Working group findings were broken down into categories of what works and what does not work to inform modifications to the prototype. This latter phase led to a major shift in the purpose and design of the prototype, validating the importance of using iterative codesign processes.

**Conclusions:**

Interpretive descriptive methods allow for an understanding of user experiences of patients with CCDD, their carers, and primary care providers. Qualitative methods help to capture and interpret user needs, and identify contextual barriers and enablers to tool adoption, informing a redesign to better suit the needs of this diverse user group. This study illustrates the value of adopting interpretive descriptive methods into user-centered mHealth tool design and can also serve to inform the design of other eHealth technologies. Our approach is particularly useful in requirements determination when developing for a complex user group and their health care providers.

## Introduction

### Overview

Software developers have historically relied on standard processes for requirements determination and functional specification [1,2]. Commonly used development models range from the conventional waterfall approach (ie, sequential design process) [3] through to more contemporary methodologies such as agile development (ie, frequent testing of iterative designs) [4]. The evolution in methods for functional specification have largely focused on finding ways to mitigate the challenge created by user or environment requirements that evolve during the development lifecycle. Iterative development approaches such as prototyping emerged to address difficulties experienced in identifying user requirements.

Almost all software requirement elucidation techniques assume the ability to engage—at specific stages or in all stages in the software specification process—with cognitively and physically able user populations. Less well understood is how to successfully elicit user specifications from medically fragile populations, such as those with complex chronic diseases and disabilities (CCDDs) [5-7]. These are individuals with two or more chronic conditions (ie, multi-morbidity) and who often face social, environmental, and contextual issues that impact on their health care needs and ability to manage [8]. These patients are among the heaviest users of the health system [7], and thus increasingly garner the attention of insurers—public or private—who are searching for cost-effective solutions [9]. At the same time, patients are seeking to minimize the burden of their illness as they navigate their way through a highly complex health care system.

In today’s technology-enabled world, there has been a plethora of patient-centered apps, online medical resources, wearable fitness technologies, and similar tools to promote adherence to treatment plans [10-13]. What is strikingly absent are information-enabled solutions designed specifically for high users of the health system [14], like patients with CCDD. The majority of apps are focused on single-disease management, thereby not aligned with the needs of complex patients [15]. Furthermore, development methodologies used in the creation of eHealth solutions lack the ability to embrace users with CCDD in the specification process.

We posit that eHealth solutions that target CCDD patients are needed, and that the success of these solutions will in part be determined by incorporating persons with CCDD in the development process. We adapted a conventional software development process to make CCDD patient users central to the specification and testing process. Using a multi-phased user-centered approach, we sought to build, deploy, and test a patient-centered app to improve quality of care and patient experience for patients with CCDD in primary health care settings. This paper describes the development phase in the creation of the electronic Patient-Reported Outcomes (ePRO) tool, with particular attention to the use of qualitative methods incorporated into the software specification process to facilitate user feedback from CCDD patients—a user population for which traditional design approaches may not be well tailored. Tool development phases were supported by the technology partner, QoC Health Inc.

### Background and Significance: Adopting Qualitative Methods in a User-Centered Design Approach

Until the mid-1990s, most software development methodologies were anchored in a linear process based on the premise that the more detailed the specification, the greater the prospect of realizing a well-functioning solution. The System Development Life Cycle [16], IBM’s Joint Application Design (JAD) [17,18], and later the Rational Unified Process [19] were typical of the era. As the opportunities to “computerize” increasingly complex systems grew, development methodologies shifted toward increasingly iterative development processes, with agile development being an excellent example of a contemporary approach [4]. Central to these approaches was the concept of user-centered design [20].

User-centered technology development is “characterized by a focus on the user, and on incorporating the user’s perspective in all stages of the design process” [21, p 1]. This approach is often iterative, involving multidisciplinary design teams, and emphasizes the need to incorporate user feedback as part of the design, testing, and implementation process [21]. Previous studies have noted that adopting user-centered design approaches can improve usability and implementation [22] and can also improve user acceptance and satisfaction with new systems overall [23]. Iivari and Iivari [24] suggest that there are four dimensions of user-centeredness: user focus, work-centeredness, user involvement or participation, and system personalization. One or more of these dimensions may be the focus on the user-centered method employed, and it is suggested that developers and researchers strive to include all four in the design approach. Also important is attention to the provision of appropriate processes and supports to encourage meaningful engagement and empowerment by users involved in the design process [25].

Design evaluation approaches suggest the use of rigorous research methods and evaluations in order to support capturing and incorporating user input [26]. We looked to combine rigorous qualitative research evaluation with an iterative design approach to obtain user feedback to develop a mobile solution that will meet the needs of patients and their primary care providers. Qualitative methods that capture individual experiences and perceptions [27] provide a useful toolkit to a user-centered design evaluation approach. Interpretive description, a form of qualitative inquiry, draws on data collected through in-depth interviews and focus groups to capture human experience [28]. Understanding the care experiences and needs of patients with CCDD requires attention to the context in which their physical and mental health and social needs are intertwined [8]. Qualitative inquiry captured in an open setting closer to participants’ lived experience allows us to capture the complexity and breadth of experiences of patients with CCDD, which may not be possible through typical user-centered design approaches, such as one-on-one, lab-based walk-throughs [29], which capture information—sometimes both qualitative and quantitative—in a closed setting. Iterative changes to the tool can be informed by rich user experience data—ensuring user focus—supporting user involvement in the design and development of a truly person (user)-centered tool.

This paper describes the design and development approach to create the ePRO tool, specifically the process of incorporating qualitative research methods into user-centered design approaches. Key lessons learned are offered as a guide for other eHealth and mHealth research and technology developers working with complex patient populations and their primary care providers (PCPs).

### The Electronic Patient-Reported Outcomes Tool

In this section, we offer a brief description of the ePRO tool that was developed through the process described in this paper. The ePRO tool includes a portal system for providers and patients to set up and monitor goals, as well as a mobile device for patients to track their goals. There is also a Hospital CheckOut feature that allows patients to report when they have visited and been discharged from a hospital—primary care providers see this information when they access the portal. To set up goals, patients and providers collaboratively work together on the portal system during an in-person visit. After initial set-up, both patients and providers can view patient progress on the portal any time in between visits. Patients also have the option of inputting their monitoring data on the portal if they do not wish to use their mobile device. A full description of the new tool will be published in our forthcoming paper outlining our usability pilot (see Multimedia Appendix 1).

## Methods

### Overview

**Figure 1:**
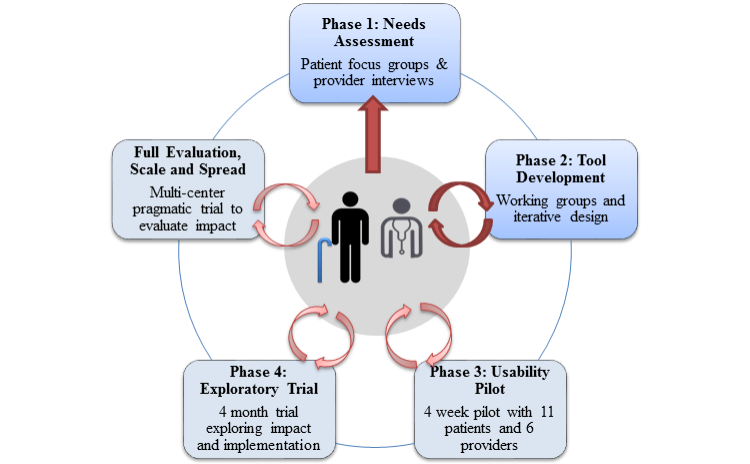
Development approach.

Guided by user-centered design principles [21] and other studies involving the development of mHealth technologies [30-33], we adopted a multi-phased research approach to support the iterative design and development of the ePRO tool. Full ethics approval was obtained from the Joint Bridgepoint Hospital-West Park Healthcare Centre-Toronto Central Community Care Access Centre-Toronto Grace Health Centre Research Ethics Board. At the outset, we broadly intended to develop an mHealth solution to support community-dwelling patients with CCDD and their PCPs. Figure 1 provides a visual depiction of our design and development method.

As depicted in Figure 1, patients, carers, and PCPs were involved at each step in the design process. This paper describes Phases 1 and 2 with specific emphasis on provider input, as shown in Figure 1. Methods and findings from the patient-focused aspects of Phase 1 have been published elsewhere [34,35].

The tool was developed within a primary health care practice, which included an interprofessional team of PCPs composed of physicians, nurse practitioners, registered nurses, social workers, and dietitians. Feedback on the tool was captured from PCPs who varied in their interest and willingness to engage in new technologies. Two PCPs expressed low interest and willingness in the working group, while the rest were more keen to try new technologies as part of care delivery.

### Phase 1: User Needs Assessment—Provider and Expert Input and Response to Patient-Identified Needs

Phase 1 consisted of focus groups with patients and carers plus interviews with content experts (CEs) and PCPs from the practice. This phase of development was mainly concerned with ensuring a user focus and capturing work-centeredness dimensions of user-centered design in that we sought to understand users' needs and the tasks needed to address those needs. Focus group findings in Phase 1, along with a literature review [34], provided the initial building blocks for tool development. A total of 14 patients and carers participated in four focus groups in the fall of 2013. Of the 14 participants, 10 (71%) were patients, 2 (14%) were caregivers only, and 2 (14%) were both patients and caregivers. The participants' average age was 64 years (range 42-90), and 9 out of 14 (64%) participants were female. Patients participating in the focus groups reported multiple chronic illnesses, including diabetes, chronic pain, osteoarthritis, osteoporosis, anemia, cardiac conditions, glaucoma, and mental illness [35]. Initial findings from the focus groups and available resources suggested that the tool should support better communication between patients and PCPs around three key areas:

1. Information about symptoms and functional status (ie, pain, mobility, depression/anxiety, activities of daily living [eg, bathing, toileting], and social well-being).

2. Medication management support (ie, reminders, renewals, and reporting side effects).

3. Educational materials and/or trusted websites to support self-management.

Next, purposive sampling [36,37] was used to identify PCPs and CEs who could provide the feedback required to refine the tool. Semistructured interviews were conducted with the PCPs, as well as CEs, in at least one of the fields of development or utilization of eHealth and/or research or service delivery experience with CCDD patients. CEs were identified through their academic, clinical, and/or research networks.

PCPs were selected from the primary care practice where the tool would be piloted and tested. These PCPs had been engaged in the project from early stages and had attended several meetings to receive updates on the project. A patient advocate was also interviewed, as this individual is experiencing CCDD who has engaged with other eHealth technologies as part of their care, and has previously served as a patient representative in other research projects.

PCPs and CEs were given a summary of patient focus group findings and asked their perspectives on the following: (1) the value of ongoing monitoring of symptoms and functional status as part of usual care, (2) what types of information should be shared about those symptoms (ie, indicators, scales, and contextual information) and how it could best be shared, (3) the role of patients accessing appropriate educational materials, (4) how different communication methods would fit into provider workflows, and (5) other aspects of primary health care delivery that may be important to capture for managing patients with CCDD.

### Phase 2: Tool Development

**Figure 2:**
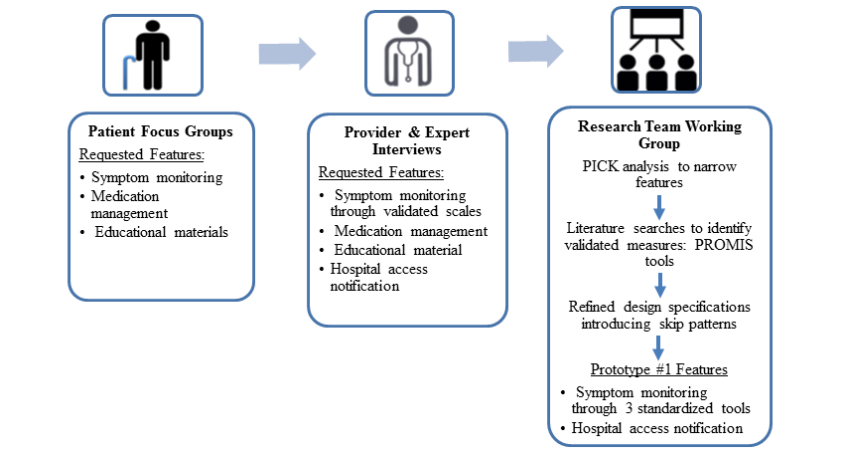
Prototype development process. The first two icons are open source from Iconfinder [38] and the last icon is open source made by Flaticon [39]. PICK: Possible, Implementable, (to be) Challenged, (to be) Killed.

Based on the Phase 1 findings, we identified four key features for the tool: symptom monitoring; medication management; educational resources; and hospital visit notification, as hospital access notification was identified in the provider interviews as an ongoing problem. Figure 2 shows the prototype development process; in the figure, the first two icons are open source from Iconfinder [38] and the last icon is open source made by Flaticon [39]. Prototype development and refinement occurred over a series of teleconferences and meetings between the research partners and the technology partner.

Patients, PCPs, and CEs who participated in the earlier stages of the study were invited to provide feedback on a working prototype during separate 2-hour working group sessions. At each working group session, participants were asked (1) whether the tool captured issues of importance to patients with CCDD, (2) whether the tool was easy to use and understand in terms of question wording and interface, and (3) whether there were other ways that we could gather similar information. The working groups aimed to support user involvement in the design process, while allowing us to pay attention to unique use cases and identify how we could build in system personalization.

The working groups consisted of modified cognitive walk-throughs [29]—participants “walk through” the sequence of tasks—in order to test the usability of the system. In contrast to one-on-one cognitive interviews, working groups provide an opportunity for individuals to engage in open dialogue and reflect on their diverse personal and professional experiences allowing us to effectively capture the breadth of multiple user experiences. Each session was audiotaped and recordings were used to verify and substantiate researcher notes taken during and after the groups.

### Data Analysis

Data analysis was conducted by reviewing notes and transcripts from interviews and working groups. An iterative analysis technique was used in which data were reviewed by two researchers, first independently and then together at multiple points aligned with the stage of tool development. Findings were organized in relation to the tool design and function to help systematically inform modifications to the tool. We used an interpretive descriptive approach in which findings are compared to a starting point [28]—the ePRO tool and prototype. The tables were written up into a summary report for Phase 1 (PCP and CE interviews) to inform the development process described above, and another for Phase 2 (prototype development process) to inform additional tool changes to be made prior to Phase 3 (usability pilot to be described in a future manuscript).

## Results

### Participants

Table 1 offers a description of the participants in Phases 1 and 2. Patients and carers who participated in the Phase 2 working groups had also attended the Phase 1 focus groups. Although more patients and caregivers from Phase 1 were invited to participate in Phase 2, many were unable to attend due to health issues.

### Phase 1: Primary Care Provider and Content Expert Interviews

PCP and CE interviews ran for approximately one hour. PCPs and CEs identified that symptom monitoring, medication management, and educational resources were all important aspects of care delivery for patients with CCDD. All supported the idea of ongoing monitoring of pain symptoms and mobility in relation to activities of daily living, as well as anxiety and depression symptoms. PCPs and CEs identified a variety of symptom-related variables and scales with little consensus except for the need for validated measures. PCPs identified the need to know when their patients were admitted to a hospital, as they frequently did not know when this had occurred, resulting in little or no follow-up and poorly coordinated care.

### Phase 2: Prototype Development Process

Figure 2 depicts how the user needs assessment was used to inform the iterative prototype development process. As can be noted in Figure 2, two features were removed through the design process by engaging in a PICK analysis—PICK stands for Possible, Implementable, (to be) Challenged, (to be) Killed. A PICK analysis is a lean technology development process in which features are assessed in terms of the time and resource investment required, and the anticipated value added [40]. The assessment allowed us to determine whether a feature was (1) Possible (ie, easy to accomplish but with a low value add), (2) Implementable (ie, easy to accomplish with a high value add), (3) to be Challenged (ie, was difficult to accomplish but had a high value add), or (4) to be Killed (ie, difficult to accomplish with a little value add). Our focus was on identifying features that fell into the *Implementable* category. The medication management and educational features were challenged, as they were difficult to accomplish given our resources and time frames. In addition, our technology partner was developing similar features for another project, thus the features could be incorporated into the ePRO tool in a future iteration.

The prototype included two features: symptom monitoring and hospital access notification. Symptom monitoring focused on symptoms identified as most important in the patient focus groups—pain, mobility, anxiety/depression, and social well-being [35]. A literature review revealed three Patient-Reported Outcome Measurement Information System (PROMIS) tools—the Global Health Scale, the Pain Interference Scale, and the Improved Health Assessment Questionnaire—as appropriate to needs as they captured symptoms of interest and were validated in chronic disease populations [41-43]. To minimize respondent burden, skip patterns were created so patients could only answer scales that were relevant to their current symptoms. A free-text comment box was included at the end of the symptom reporting to allow patients to provide contextual information to meet the needs of patients who wanted their providers to understand them as “whole persons” [35].

The hospital visit notification feature allowed patients, and/or their carers, to notify the PCPs when the patient had been to an emergency department or admitted to hospital, including the name of the hospital, date of admission, and reason for admission. Once notified, PCPs could request discharge reports from hospitals, which are not typically received in a timely manner.

### Working Group Findings: A New Direction

The three working groups provided rich and valuable feedback on the prototype. Findings were separated into categories of *what works* and *what does not work*.

#### What Works

##### Functionality: Ongoing Monitoring and Tracking

All three groups felt the remote monitoring function was valuable. Patients appreciated being able to see changes in their symptoms over time. PCPs felt that having information about their patient’s symptoms over time could enable them to see what specific issues their patients had been facing and then target discussions at the point of care to those issues.

##### Content: Including Contextual Information

All three working groups saw value in capturing contextual information through the use of open-ended questions in order to provide a more well-rounded understanding of patients' symptoms and capacity to self-manage.

#### What Does Not Work

##### Issue 1: The Length of the Tool

All three groups felt that the monitoring questions were overly burdensome. For patients, this meant potentially taking 20-25 minutes once a week to answer questions, and for PCPs it entailed sifting through a large amount of monitoring data.

##### Issue 2: Fitting in With Provider Workflows

While PCPs saw the potential for symptom monitoring between visits, they were uncertain how they would fit monitoring into their daily schedules. PCPs also had concerns about liability issues, if they were to be responsible for monitoring a large number of patients.

##### Issue 3: Unclear Answer Keys, Scales, and User Flow

There were concerns from all three groups regarding confusing visual analog and Likert scales. Patients expected a rating of 10 to be good; however, the scales were based on tools designed for providers, who tend to see high values (ie, spikes) as a negative health outcome. Additionally, some of the scales were flipped. A high number would be a good outcome for one question but a bad outcome for another. It was felt that standardization in line with the preference of the patients would improve usability of the tool.

**Table 1:** Study participants.

Participants	Details	Type of group in each phase	
		Phase 1	Phase 2
Patients	9 female Average age=64 years (range 42-79) 2 patients were also caregivers All identified have two or more chronic conditions they find difficult to manage	Focus group (n=12)	Patient/caregiver working group (n=4) 3 females, 1 male Of these, 1 female patient was also a caregiver
Caregivers	All female	Focus group (n=2)	Patient/caregiver working group (n=1)
PCPs ^a^ (n=7)	General practitioners (n=2)	Interview	Provider working group
	Nurse practitioner	Interview	Provider working group
	Registered nurse	Interview	Provider working group
	Dietitian and diabetes educator	Interview	Provider working group
	Administrative staff member	Interview	Provider working group
	Executive director	Interview	N/A^b^
CEs ^c^ (n=6)	Complex pain	Interview	Expert working group^d^
	eHealth	Interview	N/A
	eHealth for chronic conditions	Interview	Expert working group
	Rehabilitation in complex populations and PROs^e^	Interview	Expert working group
	Complex stroke	N/A	Expert working group
	Complex patient	Interview	Expert working group

^a^PCP: primary care provider.

^b^N/A: not applicable.

^c^CE: content expert.

^d^Research team participants in expert working group (n=4).

^e^PRO: patient-reported outcome.

##### Issue 4: Content Issues

The wording of some questions, including length, reading level, double-barreled questions, and negative labeling connotations for some questions—particularly around mental health—were seen as problematic by patients. Providers also wanted to see questions on patient confidence and self-efficacy included, which were subsequently added to the tool.

### Shifting From Monitoring to Supporting Self-Management and Patient-Centered Delivery

The issues identified by all participants suggested the need to rethink the utility and purpose of the tool moving forward. Provider workflow concerns, the length of the tool, and content issues suggested very low usability of the prototype. More importantly, working group findings revealed that much of the symptom data of importance to patients were related to the types of care planning and goal setting that the PCPs would engage in with patients. This realization prompted a major shift in the purpose of the tool away from an application that only captures patient-reported outcome measures to a tool that actively uses those measures to improve the design and delivery of goal-oriented primary health care while supporting self-management for patients with CCDD.

Goal-oriented models of care can support improved patient-centered care delivery [44], identified as a crucial need via the patient focus groups. Furthermore, a goal-oriented approach to care aligns with the patients’ desires to improve their health status with respect to symptom management. Goal setting has been identified as an important process to improve care for complex and chronically ill patients [44,45]. Despite this, goal-oriented care has proven to be a challenge in primary care settings [46] and goals are often not agreed upon between providers and their complex patients [47].

## Discussion

### Implications for Development

#### Overview

An interpretive descriptive approach allowed us to capture diverse and unique user needs of a complex and often overlooked patient population. Our data collection and analysis strategy informed a major shift in system requirements which may have been missed if we had relied solely on more traditional requirement elucidation techniques. The discussion will outline three key lessons learned with regard to developing mHealth tools for patients with CCDD in primary health care settings, and examine how the use of interpretive descriptive methods helped address and respond to these issues. These lessons could extend to development of broader eHealth technologies as well.

#### Lesson 1: Developing Tools for Patients With Complex Chronic Diseases and Disabilities Requires Balancing Multiple and Diverse Needs

A core challenge in adapting functional specifications methodology for use by people with CCDD was the difficulty associated with balancing the needs of multiple diverse end users. Although the initial intention was to create a patient-centered tool, there had to be acknowledgement of the needs of PCPs who would be engaging with the tool. While the original prototype was meeting patient needs for symptom monitoring, it did not align with provider workflows. Qualitative methods enabled us to capture why individuals responded as they did, including contextual factors that played a role in that response, and allowed for probing at ways to reconcile diverse needs. Codesigning interventions with CCDD patients and providers has already been noted to be key to success [48,49]—an approach that should extend to developing other types of mHealth and eHealth interventions as well.

Other studies have noted the need to engage with users to further refine the purpose and goals of new information technology (IT) systems [30,32]. For example, in designing the Coplintho project, De Rouck and colleagues underwent extensive early development work to identify the appropriate user groups and user needs, as well as to create a final purpose or *use case* for their tool [50]. As is the case in developing the ePRO tool, the Coplintho project drew heavily on qualitative methodologies and the designers concluded that their methods helped to refine project goals and tool functionality.

#### Lesson 2: The Purpose and Intention of the Tool Should Remain Flexible Through the Development Process

The purpose of the tool had to be flexible in order to meet user needs. Part of the challenge was that there was uncertainty regarding the specific needs of the CCDD patient population, thereby creating a number of design and development challenges, as well as research challenges, since many early discussions around the tool were exploratory in nature. Flexibility allowed for responsiveness to user needs and contexts but resulted in challenges in the research and development process that required resources to assess and continually explore different system capabilities, functionalities, and content.

#### Lesson 3: Careful Attention Needs to be Paid to Provider and Organizational Barriers to mHealth Tool Adoption

Close attention also needed to be paid to organizational and system realities in which our tool would be adopted. The organizational culture around adopting new technologies required resources in terms of IT support for PCPs. Further, provider time and workflows factored into the provider response and willingness to engage with the tool during development. Through interviews and working groups, these important contextual factors were identified and could be addressed in redeveloping the prototype.

Provider and organizational-level barriers to adopting mHealth technology are well documented in the literature. In a systematic review of factors affecting mHealth adoption, provider-level issues such as perceived usefulness and impact on patient care, and organizational-level factors such as impact on provider workflows and interprofessional communication, were found to be important [51]. It has been recommended that development stages should involve clinical perspectives in order to mitigate barriers and ensure appropriate functionality [14]. If only patient needs were considered, the ePRO tool would serve solely as a monitoring tool, which would have been unsustainable in the long term.

### Limitations

Blending qualitative specification elucidation into traditional prototyping methods is time and resource intensive and, as such, we were only able to go through a single round with a high-fidelity prototype which was “an incomplete but essentially executable version of the final product” [52, p 666] . Future studies involving multiple and diverse end users should build in additional time and funding, and potentially use lower-fidelity prototypes like wireframes to enable multiple iterations and cycles of design. Another limitation was the separation of patients and PCPs at the working group stage in order to minimize perceived power differentials that might result in patients not fully expressing their views. This concern may make codesign with both parties in the room difficult [49]. We also had a relatively low number of patients in our working group due to medical frailty. When working with a complex patient population, strategies are required to address the high likelihood of dropout, for instance, recruiting a much larger number of patients for the focus group then would be typically done. Another consideration working with this population is there may be concerns with capturing typical socioeconomic data, such as education, occupation, and household income, which could deter patients from participation. As such, we opted to forgo capturing this information at this stage of development, so as to reduce barriers to participation.

As we move forward with this work, we must also be aware that many individuals within the complex patient population, particularly older adults and those with lower household incomes, may not have their own mobile phones. To address this issue, we will make mobile phones available to participants of our pilots and trials, and make it possible for caregivers to respond on the patient;#8217;s behalf. As noted earlier, patients can also input data via a portal system, which they can access from a home computer or open access computers at libraries.

### Conclusions

The use of interpretive descriptive methods can be of particular use in requirements determination and functional specification in software development for diverse populations, such as patients with CCDD. Qualitative methods can do the following: help to ensure all end users' needs are captured and understood within their unique contexts, allow for a flexible purpose of tools in the early stages of design, and help to capture contextual enablers and barriers to the tool’s uptake. The key lessons that have emerged from the process of developing the ePRO tool can be adopted by other researchers or developers of mHealth and eHealth technologies. Using interpretive descriptive qualitative methods as part of a user-centered design was pivotal to capturing, analyzing, and implementing user feedback. To help generate a better understanding between patients and providers of each other’s experiences, future design stages will seek to bring patients and providers together to align with established codesign methods [52].

## Appendix

Multimedia Appendix 1 Electronic Patient-Reported Outcomes (ePRO) tool screenshots.

## Abbreviations

CCDD: complex chronic disease and disability
CE: content expert
ePRO: electronic Patient-Reported Outcomes
IT: information technology
JAD: Joint Application Design
N/A: not applicable
PCP: primary care provider
PICK: Possible, Implementable, (to be) Challenged, (to be) Killed
PRO: patient-reported outcome
PROMIS: Patient-Reported Outcome Measurement Information System
