# Supplementary material for: Improving Patient Experience and Primary Care Quality for Patients With Complex Chronic Disease Using the Electronic Patient-Reported Outcomes Tool: Adopting Qualitative Methods Into a User-Centered Design Approach
Source: JMIR Res Protoc. 2016 Feb 18;5(1):e28. doi: 10.2196/resprot.5204 (PMC4777883; doi:10.2196/resprot.5204)
Supplement: Multimedia Appendix 1 [file resprot_v5i1e28_app1.pptx]

## Slide 1
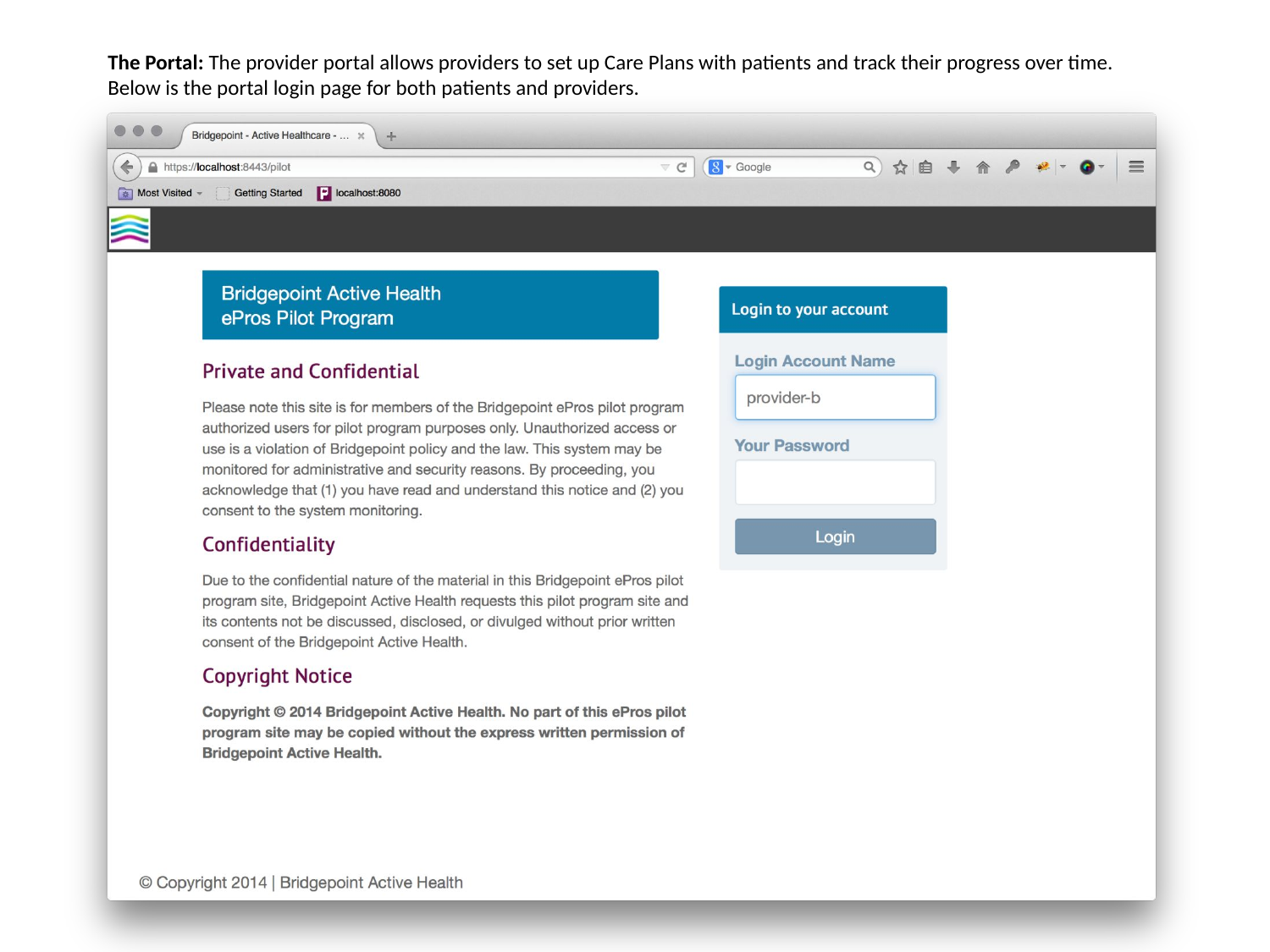

The Portal: The provider portal allows providers to set up Care Plans with patients and track their progress over time. Below is the portal login page for both patients and providers.

## Slide 2
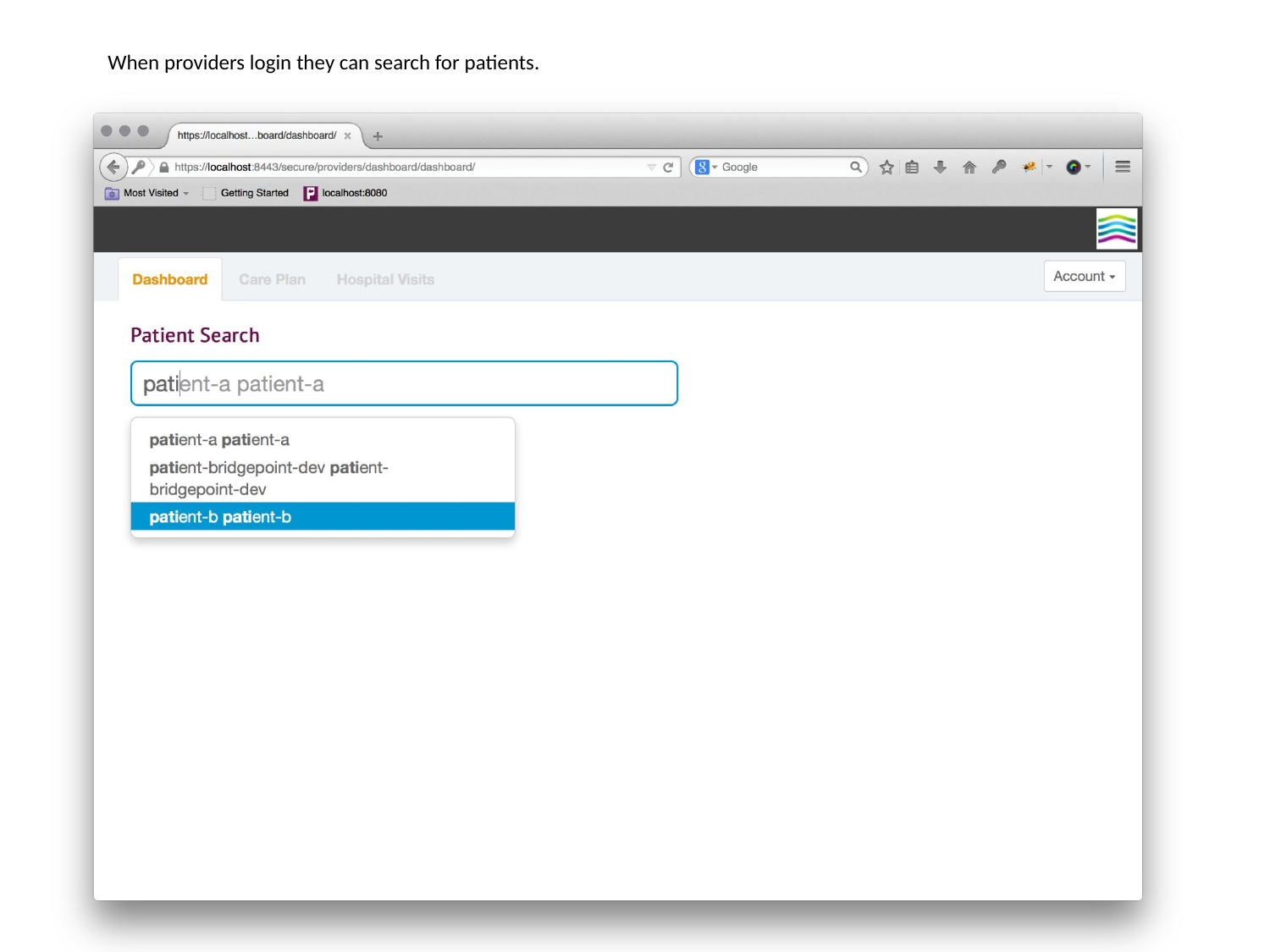

When providers login they can search for patients.

## Slide 3
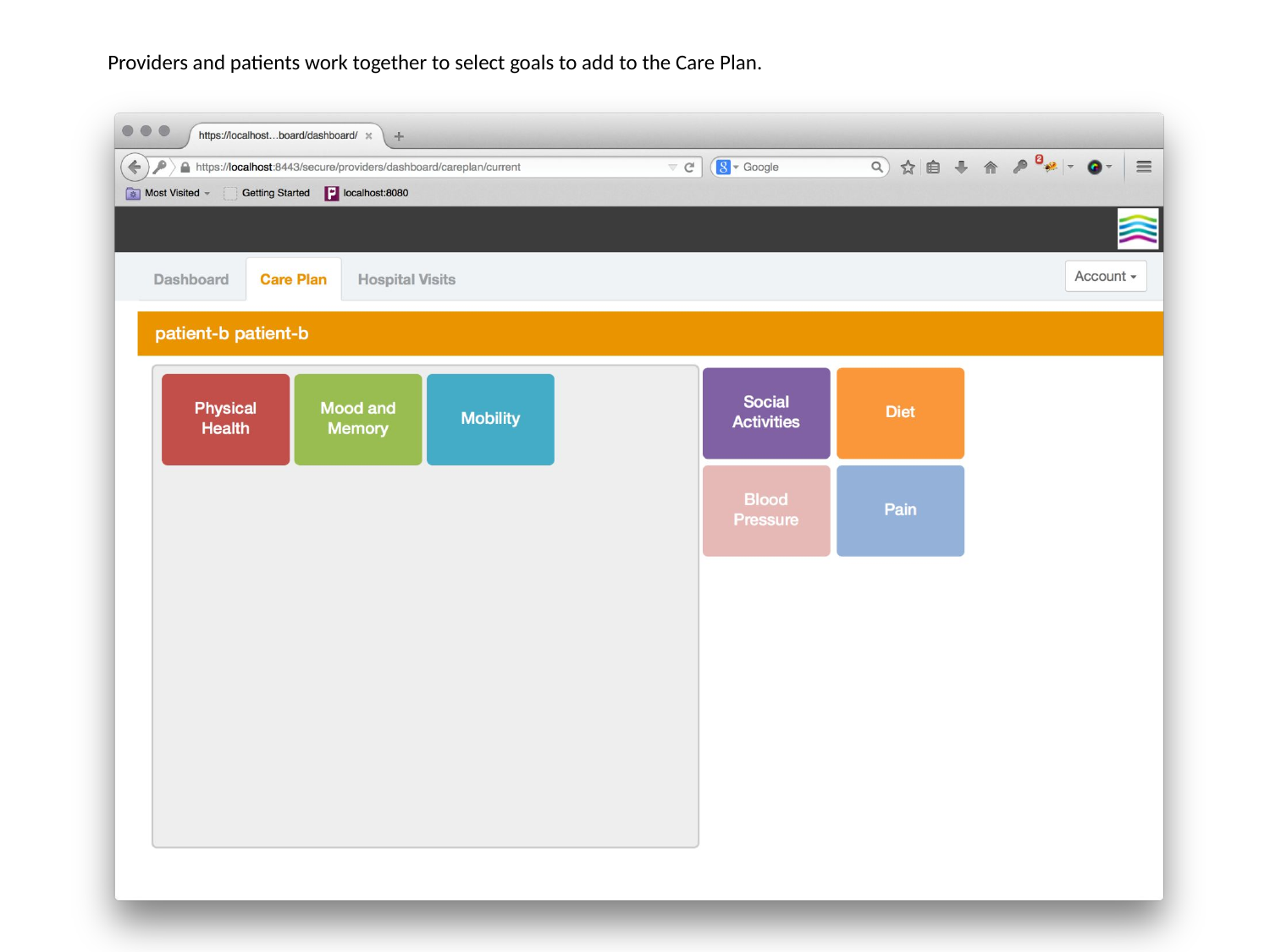

Providers and patients work together to select goals to add to the Care Plan.

## Slide 4
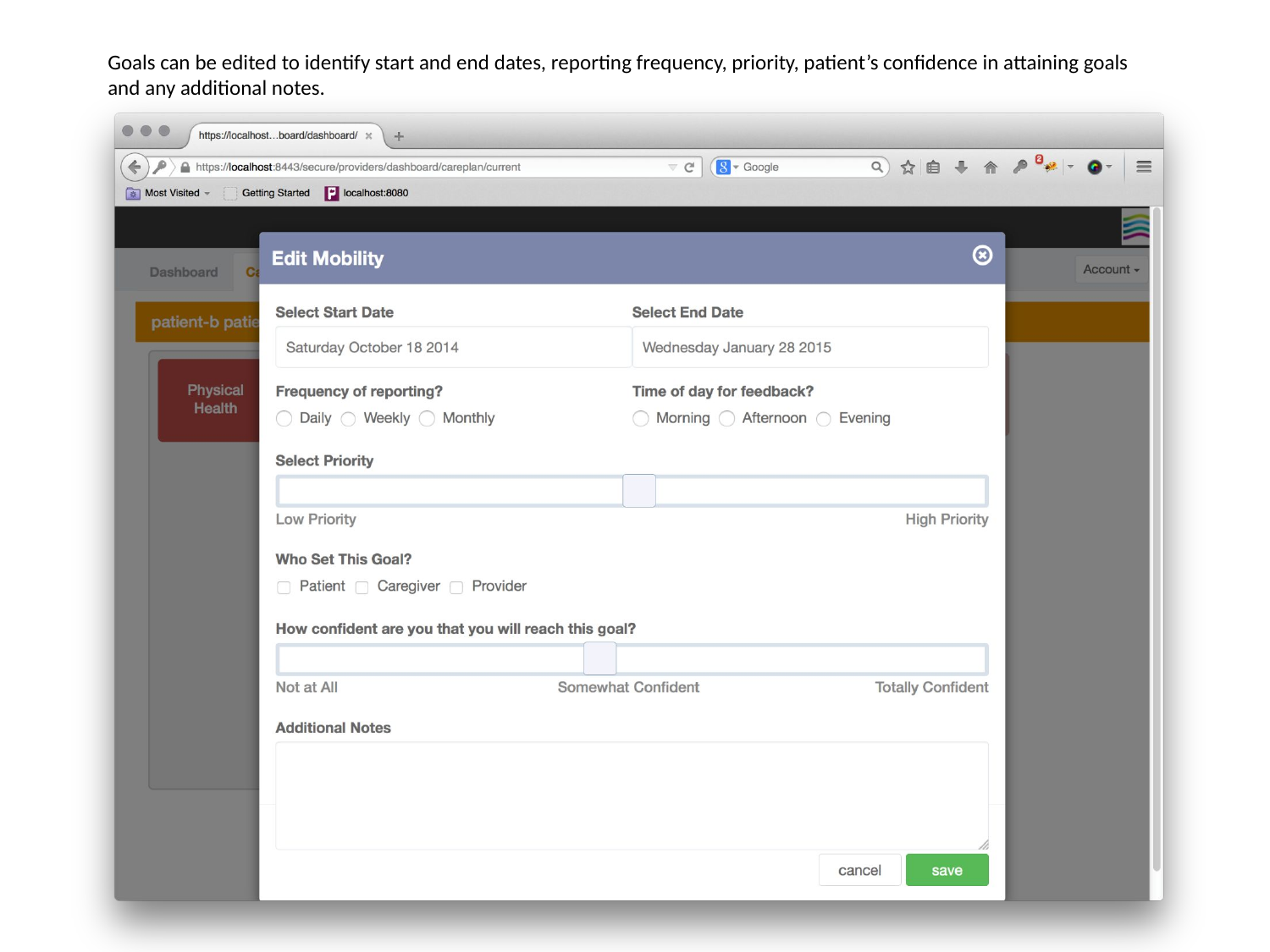

Goals can be edited to identify start and end dates, reporting frequency, priority, patient’s confidence in attaining goals and any additional notes.

## Slide 5
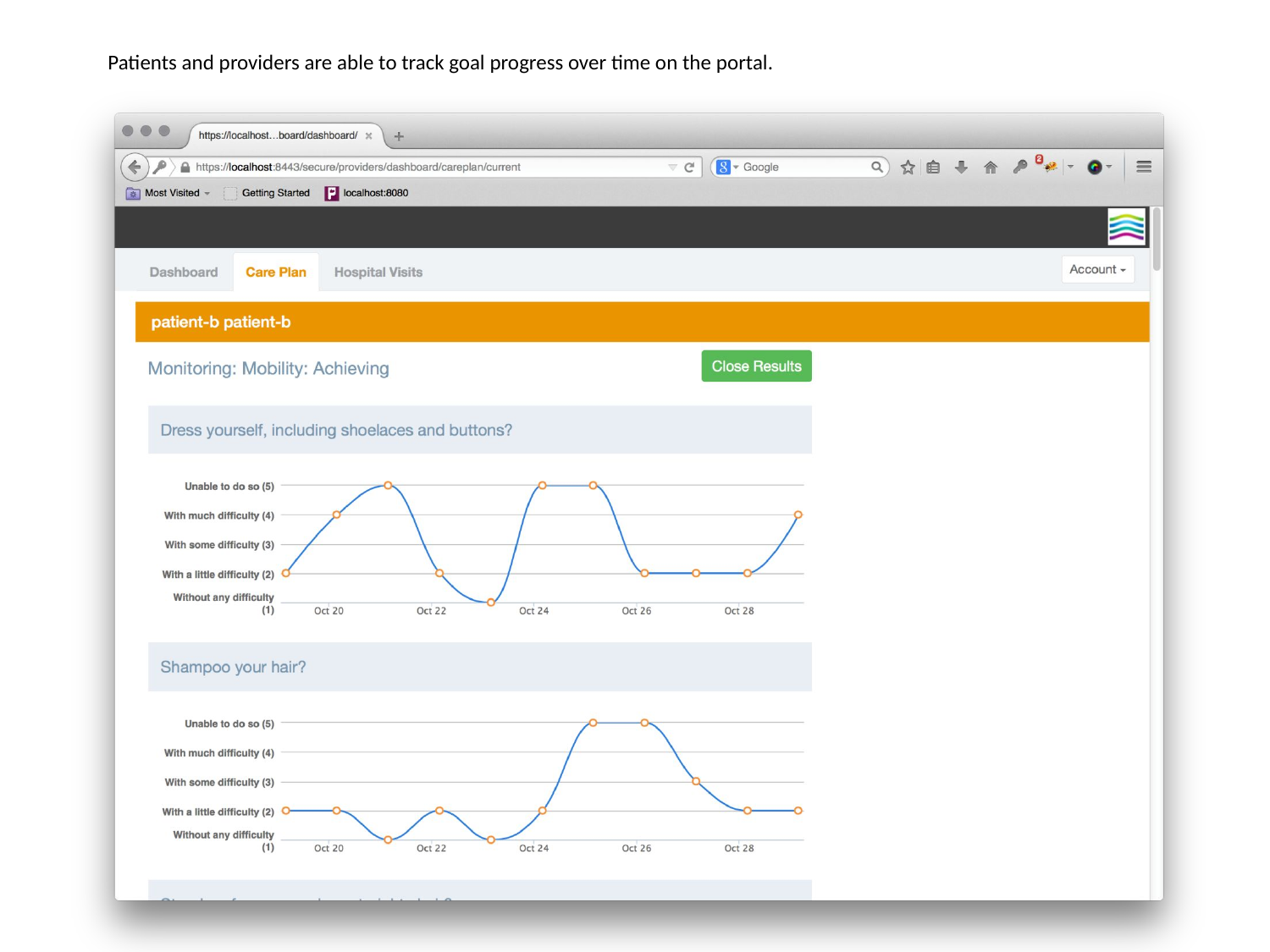

Patients and providers are able to track goal progress over time on the portal.

## Slide 6
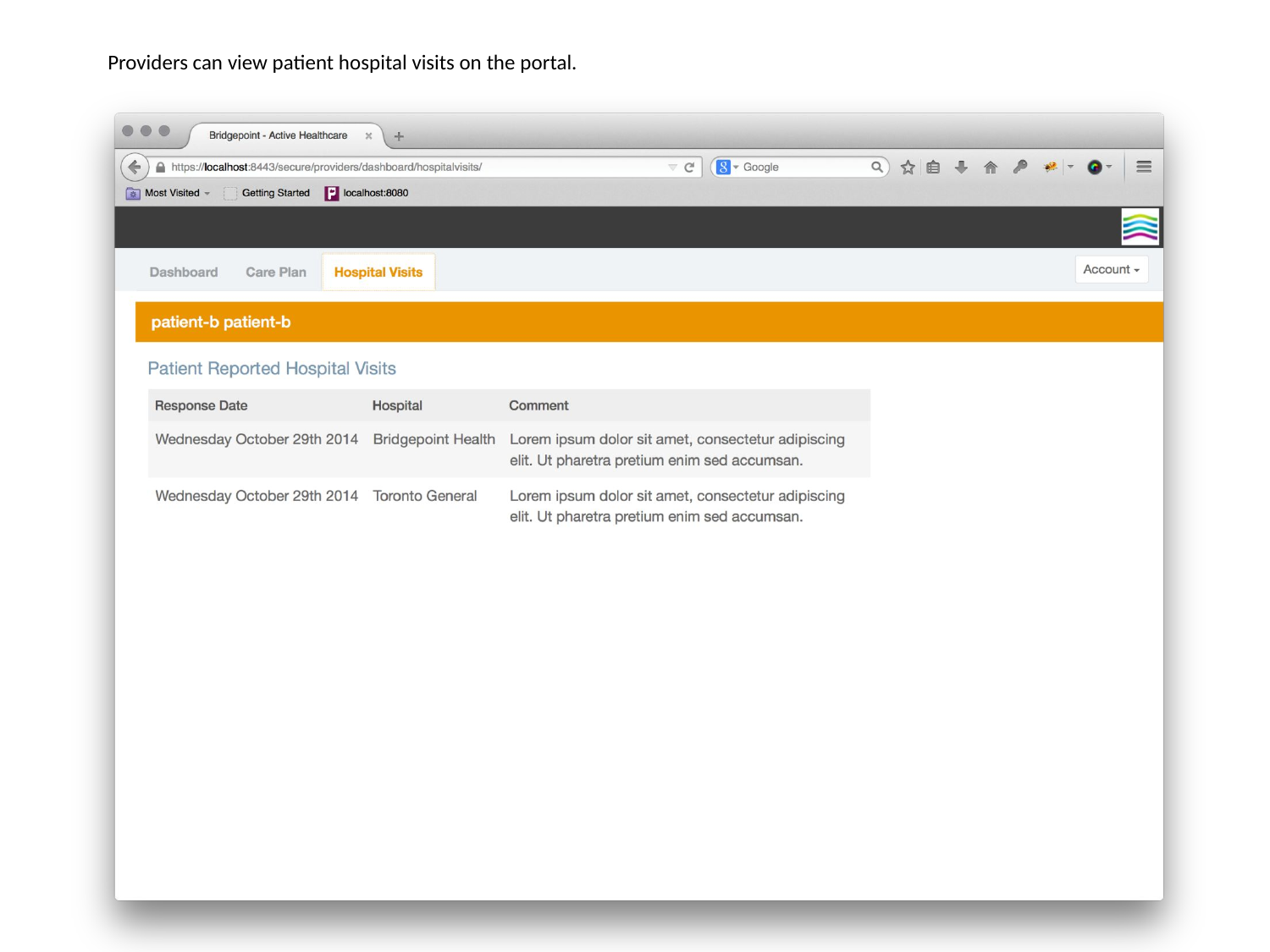

Providers can view patient hospital visits on the portal.

## Slide 7
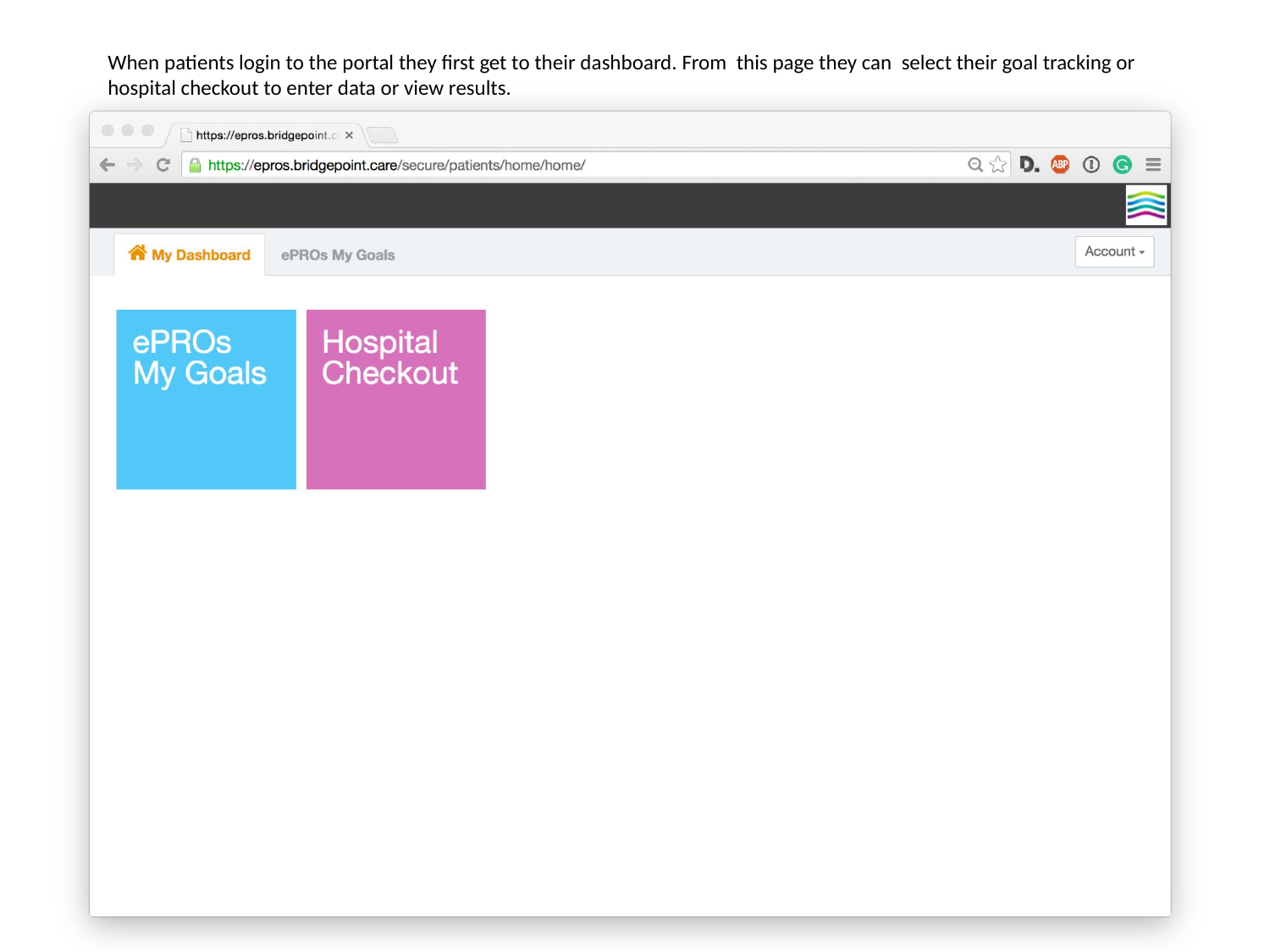

When patients login to the portal they first get to their dashboard. From this page they can select their goal tracking or hospital checkout to enter data or view results.

## Slide 8
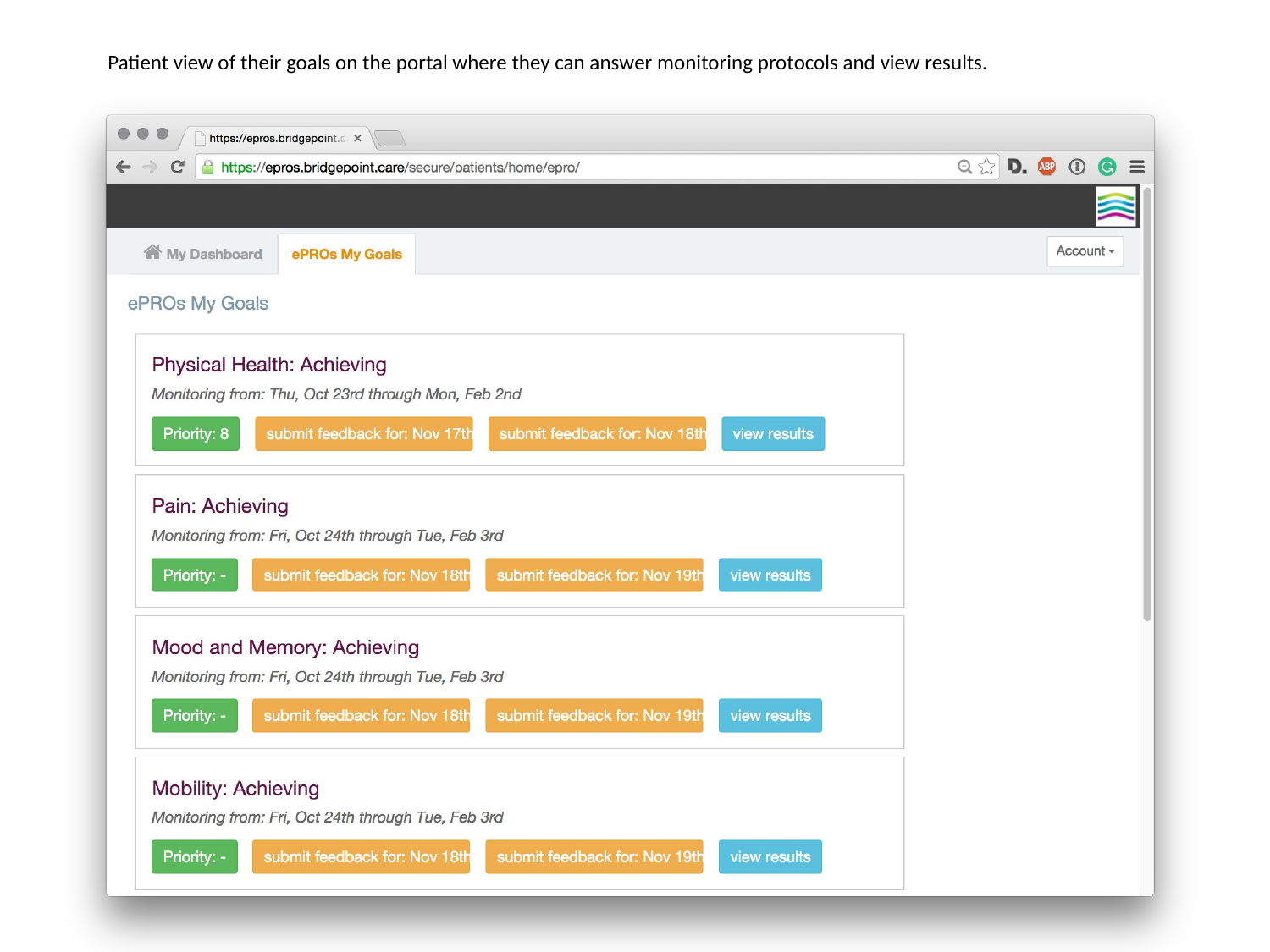

Patient view of their goals on the portal where they can answer monitoring protocols and view results.

## Slide 9
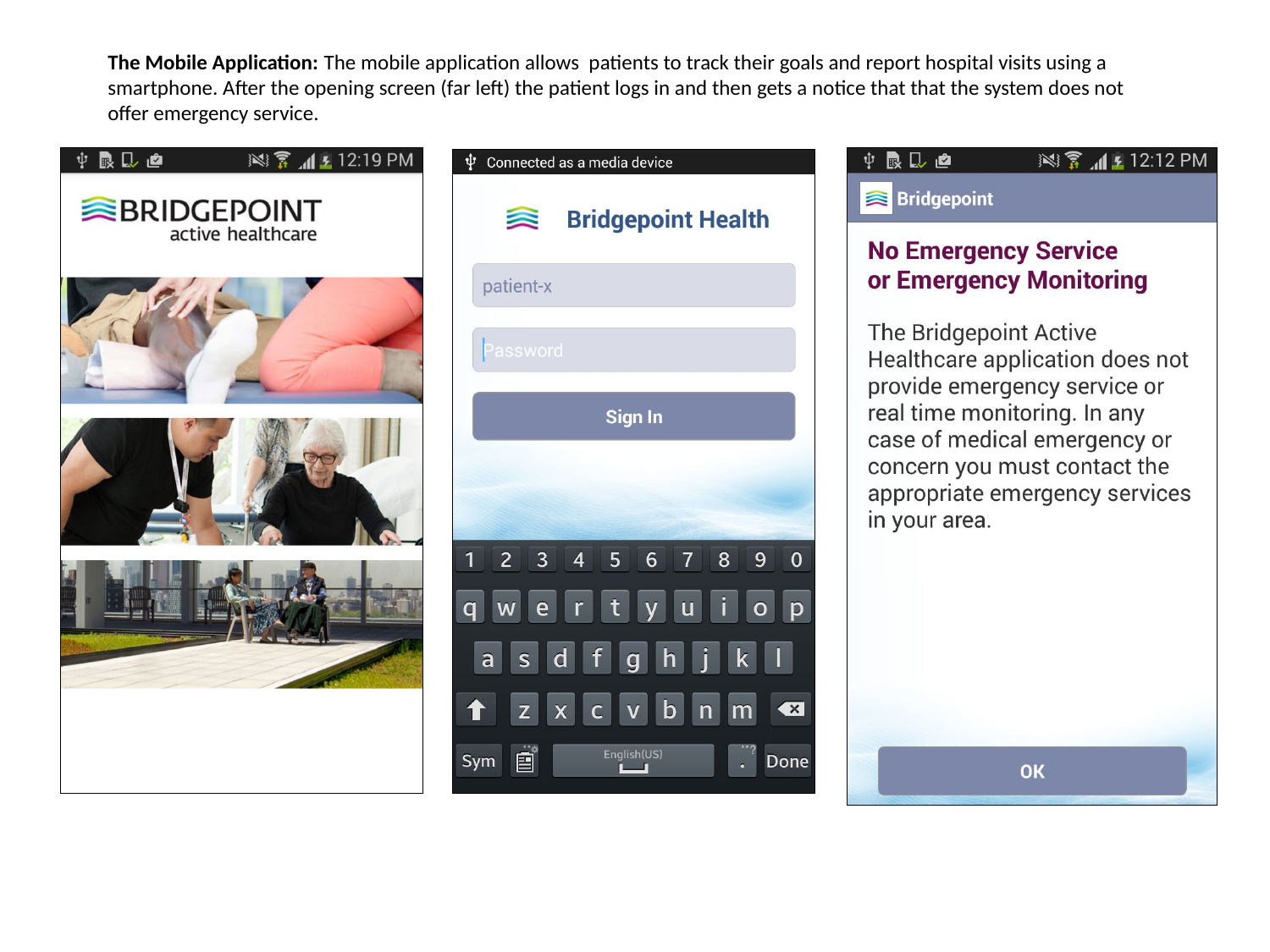

The Mobile Application: The mobile application allows patients to track their goals and report hospital visits using a smartphone. After the opening screen (far left) the patient logs in and then gets a notice that that the system does not offer emergency service.

## Slide 10
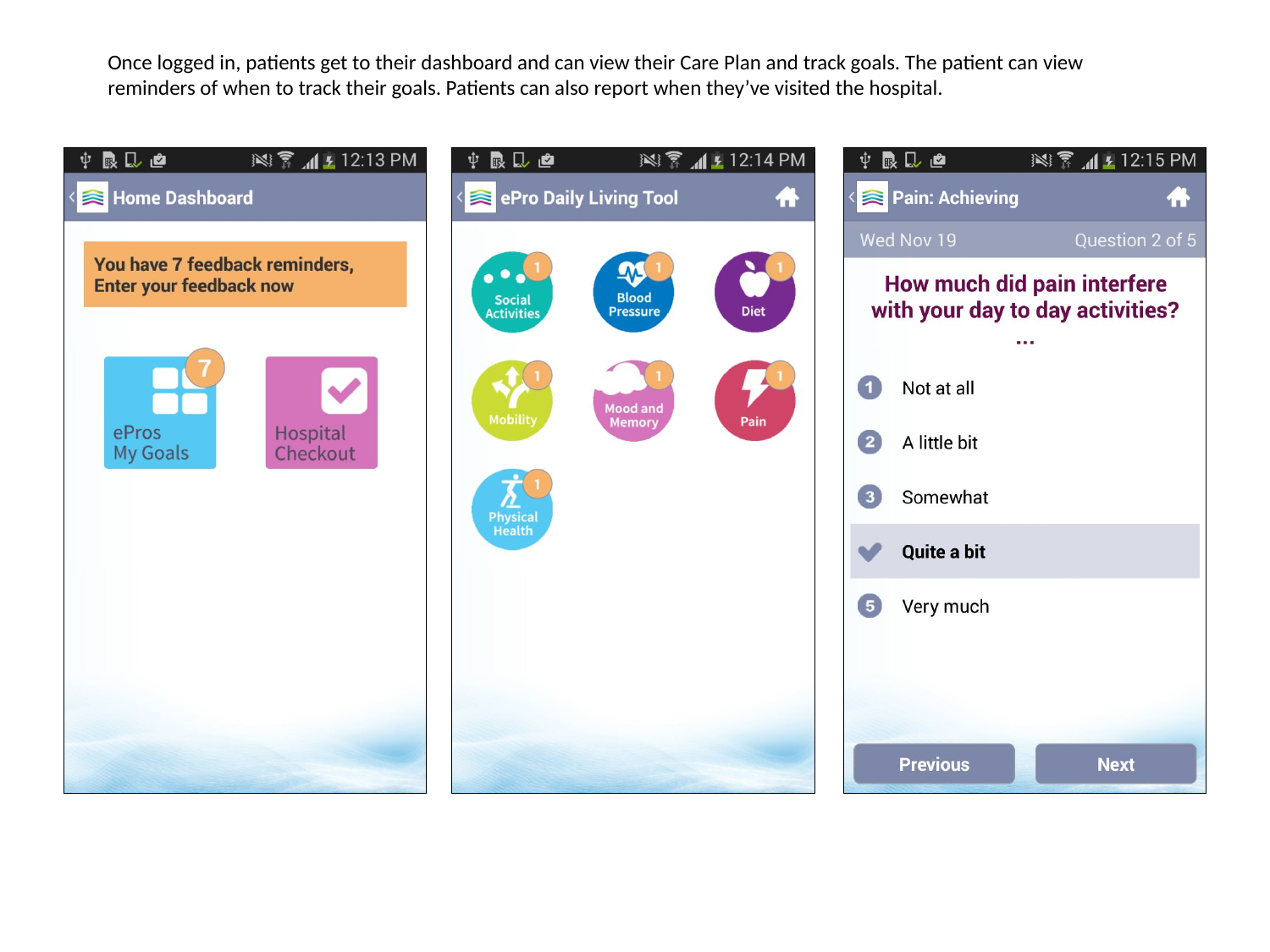

Once logged in, patients get to their dashboard and can view their Care Plan and track goals. The patient can view reminders of when to track their goals. Patients can also report when they’ve visited the hospital.
